# Association between E/e´ ratio and fluid overload in patients with predialysis chronic kidney disease

**DOI:** 10.1371/journal.pone.0184764

**Published:** 2017-09-13

**Authors:** Jae-Seok Kim, Jae-Won Yang, Jin Sae Yoo, Seung Ok Choi, Byoung-Geun Han

**Affiliations:** Department of Nephrology, Yonsei University Wonju College of Medicine, Wonju, Kang-won, Korea; Hospital Universitario de la Princesa, SPAIN

## Abstract

**Background:**

Chronic fluid overload is common in patients with chronic kidney disease (CKD) and can with time lead to diastolic dysfunction and heart failure. We investigated whether markers of fluid status, such as NT-proBNP and bioimpedance spectroscopy (BIS), can predict echocardiographic findings of diastolic dysfunction in non-dialysis CKD5 patients.

**Methods:**

BIS, echocardiography, and measurement of serum NT-proBNP were performed in patients with non-dialysis CKD stage 5 at a single study visit. E/e´ ratio reflect mean LV diastolic pressure and a ratio greater than 15 was used as a definition of diastolic dysfunction.

**Results:**

Eighty-four patients were analyzed. Forty-six patients (54.76%) had E/e´ ratio ≤15 and 38 patients (45.24%) had E/e´ > 15 (diastolic dysfunction). Patients with E/e´>15 had significantly higher serum NT-proBNP (14,650 pg/mL) than patients with to E/e´≤15 (4,271 pg/mL) and had more overhydration (OH), 5.1 liters compared to 2.4 liters. The cut-off values predicting diastolic dysfunction were found to be 2,797 pg/mL for NT-proBNP and 2.45 liters for OH.

**Conclusions:**

Regular monitoring of fluid status by BIS and NT-proBNP can be used to find patient with risk of developing diastolic dysfunction. Treatments to correct fluid overload may reduce the risk of developing diastolic dysfunction and improve cardiovascular outcome in patients with CKD.

## Introduction

Diastolic dysfunction is frequently found in patients with chronic kidney disease (CKD) and cardiovascular risk (CV) also increases as kidney function declines [1]. In hemodialysis patients, number of studies have shown structural and functional abnormalities in the heart, such as left ventricular (LV) hypertrophy, in association with fluid overload. However, few studies have extended such investigations to non-dialysis CKD [2]. Biomarkers associated with increased CV risk in CKD include left ventricular mass (LVM), carotid-femoral pulse-wave velocity, LV systolic and diastolic function, and vascular calcification. Among these biomarkers, diastolic dysfunction has been reported to be an independent predictor of mortality in multivariate analysis. Diastolic dysfunction involves abnormal LV relaxation, filling, and distensibility and is frequently found in patients with CKD [3–5]. Diastolic dysfunction may develop early in CKD [6] and is common in later stages. Thus, in one small study in patients who underwent kidney transplant, 67% were found to have diastolic dysfunction [7]. Although LV mass decreased by 23% after the transplantation, diastolic dysfunction persisted in most cases, indicating irreversibility of the structural changes, probable due to long term fluid retention and cardiac fibrosis.

Assessment and management of volume status is especially important as it can improve hemodynamic stability. Although various methods for assessing volume status including physical examination, chest X-rays, and ultrasonography are available, each method has its limitation. The sensitivity, specificity, and predictive values of laboratory studies such as NT-proBNP vary widely depending on the cut-off value, limiting their clinical utility when used alone. Recently, bioimpedance spectroscopy (BIS) performed at the bedside has gained popularity because it is a non-invasive, quick and relatively affordable method to quantitatively assess the volume status of the patient.

We investigated whether NT-proBNP and BIS are associated with the echocardiographic findings of diastolic dysfunction in patients with non-dialysis CKD stage 5.

## Materials and methods

### Patients and data collection

This study included 100 patients who were admitted to our hospital between October 2014 and July 2016 for planning a renal replacement therapy. This is a retrospective cohort analysis of data on BIS, echocardiography, and serum NT-proBNP from this visit. Patients diagnosed with acute kidney injury, malignancy, infection, liver cirrhosis, mitral regurgitation, atrial fibrillation, valvular heart disease, or abnormal LV segmental wall motion was also excluded. Two patients with left ventricular ejection fraction (LVEF) less than 45% were also excluded, as was one patient who showed clear findings of overt diastolic failure. This study was approved by the Institutional Review Board of Yonsei University Wonju Severance Christian Hospital. All participants provided written informed consent prior to the study.

### Assessment of left ventricular function

Echocardiography was performed in the harmonic imaging mode using a 3-MHz transducer and commercial ultrasound system (Vivid-7; General Electric-Vingmed, Milwaukee, WI, USA). As recommended by the Heart Failure and Echocardiography Associations of European Society of Cardiology, both conventional and tissue Doppler imaging (TDI) echocardiographic techniques were used for the evaluation of LV diastolic function [5, 8]. Transmitral inflow velocities were measured using pulsed-wave Doppler in the apical four-chamber view with the sample volume placed at the mitral valve leaflet tips. Transmitral early diastolic (E wave) velocities were measured. TDI in the apical four-chamber view was used to measure LV myocardial velocities, with the sample volume placed at the septal mitral annulus. We measured the peak early (e´) diastolic mitral annular velocity and calculated E/e´ ratio [9, 10]. The left atrial (LA) dimension was measured by 2D-guided M-mode echocardiography using the parasternal short-axis view at the base of the heart. Three LA dimensions were used to calculate the LA volume as an ellipse using the formula: LA volume = (π/6)(SA1×SA2×LA), where SA1 is the M-mode LA dimension, and SA2 and LA are measurements of the short- and long-axis with the apical four-chamber view at ventricular end-systole, respectively. The LA volume index (LAVI) was calculated by dividing LA volume by body surface area (BSA), calculated using the formula: BSA = 0.007184 × weight^0.425^× height^0.725^ (m^2^). LV internal dimensions, LV wall thickness, and LVEF, measured using the biplane modified Simpson rule, were measured according to the reported recommendations [11]. LV mass was calculated following American Society of Echocardiography recommendations as LV mass (g) = 1.04×([PWTd+SWTd+LVDd]^3^×[LVEDD]^3^)×0.8+0.6, where PWTd and SWTd are the posterior and septal wall thickness at end-diastole, respectively, and LVEDD is the M-mode LV dimension with the short axis view at end-diastole [12]. To correct for body surface area, the LV mass index (LVMI) was calculated as LV mass/BSA.

E/e´ ratio was used to determine diastolic function instead of E/A ratio because the latter is greatly influenced by hemodynamic status. Among the echocardiographic parameters, E/e´ ratio reflects mean LV diastolic pressure, and E/e´ ratio >15 is known to be an indication of diastolic dysfunction [8, 13]. Echocardiography was performed by trained specialists who were completely blinded to patient information.

### Assessment of the volume status

Bioimpedance spectroscopy using the BCM^TM^ (Body Composition Monitoring^TM^, Fresenius Medical Care, Bad Homburg, Germany) was performed prior to any dialysis treatment. BCM utilizes alternating electric currents across 50 different frequencies from 5 to 1000 kHz for the measurement of fluid status. The overhydration (OH) value can be calculated from the difference between the normal expected extracellular water (ECW) and actual measured ECW [14]. The validity of BIS in a study of healthy populations and dialysis patients has already been demonstrated in comparison to standard measurement methods [15].

### Laboratory evaluations

All blood samples were collected at the study visit. NT-proBNP was measured using electrochemiluminescence immunoassay (ECLIA) on Modular analytics E170 (Roche Diagnostics, Mannheim, Germany). The analytical measurement range for NT-proBNP was 5 to 35,000 pg/ml.

### Statistical analysis

All analyses were performed with IBM Statistics Package for the Social Science version 23.0 (IBM Corporation, Armonk, NY, USA). Descriptive statistics were described in terms of the mean ± standard deviation for continuous variables. Patients were classified into E/e´ ratio >15 and E/e´ ratio ≤15 which represents with or without diastolic dysfunction, respectively. The continuous variables were compared using Two-sample T-test whereas the nominal variables (sex, presence of diabetes, use of hypertension medication, and use of medication for diuresis) were compared using Fisher’s exact test. Pearson’s correlation analysis was used to examine correlations between E/e´ ratio and other variables. To assess independent variables associated with E/e´ ratio, we performed a multiple linear regression analysis using variables that correlated with E/e´ ratio with P < 0.05 in the univariate analysis: systolic blood pressure, NT-proBNP, albumin, uric acid, OH/ECW, and ECW/TBW. Finally, we calculated a receiver operating characteristic (ROC) curve to establish cut-off values of OH, OH/ECW, ECW/TBW, NT-proBNP and LAVI that discriminate patients with E/e´ ratio >15 from those with E/e´ ratio ≤15. Statistical significance was defined as P < 0.05.

## Results

### Patients’ characteristics

The characteristics of the study population are shown in Table 1. Patients with diastolic dysfunction (E/e´ ratio >15) had significantly higher systolic blood pressure, were less commonly treated with BP medications and had more often diabetes. The age and gender were similar. Serum parameter that was significantly different between the two groups included albumin (3.59±0.63 g/dL vs 3.24±0.51 g/dL, P = 0.006). It took an average of 28.44±81.65 days to start dialysis after the assessments for patients included in this study.

**Table 1:** Demographic findings of study patients.

		E/e´ ≤15	E/e´ >15	
Variables	Total (N = 84)	N = 46	N = 38	P-value
Age, years	60.32±10.34	59.85±9.73	60.90±11.15	0.647
Gender Men	46 (54.76%)	28 (60.87%)	18 (39.13%)	0.272
Diabetes Yes	50 (59.52%)	21 (42.00%)	29 (58.00%)	0.007
BP medication Yes	78 (92.86%)	40 (51.28%)	38 (48.72%)	0.030
Diuretics Yes	58 (69.05%)	28 (48.28%)	30 (51.72%)	0.098
BPsys, mmHg	143.24±18.72	138.33±18.94	149.22±16.83	0.008
BPdia, mmHg	78.50±11.68	78.07±12.34	79.03±10.98	0.714
eGFR, mL/min/1.73 m^2^	6.29±2.28	6.54±2.41	5.97±2.11	0.258

### Volume markers and echocardiographic findings

Fractional shortening and LVEF were not significantly different between the two groups. LAVI was significantly greater with diastolic dysfunction. NT-proBNP, which is known to reflect volume status and LV functional status, was also significantly elevated with diastolic dysfunction. Among the values obtained from BIS, parameters reflecting volume status (OH, OH/ECW, ECW/TBW) were significantly higher with diastolic dysfunction (Table 2).

**Table 2:** Echocardiographic and bioimpedance findings.

		E/e´ ≤15	E/e´ >15	
Variables	Total (N = 84)	N = 46	N = 38	P-value
NT-proBNP, pg/mL	9634.0±11723.0	4,270.5±6,276.5	14,649.6±13,020.9	0.000
LA dimension, cm	4.80±0.39	4.69±0.30	4.92±0.45	0.006
LAVI, ml/m^2^	39.18±11.79	33.78±7.79	45.71±12.56	0.000
E/e´ ratio	16.23±6.42	11.91±2.49	21.46±5.80	0.000
LVEDD, cm	5.43±0.56	5.28±0.54	5.62±0.53	0.005
LVEDV, ml	145.87±31.81	137.52±26.54	155.97±34.95	0.007
LVMI, g/m^2^	125.24±33.93	114.44±24.82	138.32±38.88	0.001
Fractional shortening, %	35.07±5.03	35.83±4.69	34.16±5.33	0.131
LVEF, %	63.54±6.72	64.80±5.84	62.00±7.44	0.056
OH, liter	3.64±3.78	2.41±3.35	5.13±3.78	0.001
OH/ECW, %	17.67±16.16	11.82±15.75	24.76±13.79	0.000
ECW/TBW	0.50±0.05	0.478±0.05	0.526±0.04	0.000
ECW, liter	17.78±4.61	16.72±4.05	19.05±4.96	0.020
ICW, liter	17.71±3.92	18.24±3.79	17.07±4.04	0.177
TBW, liter	35.48±7.61	34.96±6.99	36.12±8.36	0.491

### Univariate correlation analysis

E/e´ ratio was significantly associated with systolic blood pressure, NT-proBNP, albumin, uric acid, as well as with the BIS factors, OH, OH/ECW, ECW, and ECW/TBW (Table 3).

**Table 3:** Univariate analysis of serum laboratory tests, blood pressure, and BIS parameters in association with E/e´.

	E/e´ ratio	
Variables	Correlation coefficient	P-value
BPsys, mmHg	0.280	0.011
BPdia, mmHG	0.097	0.386
NT-proBNP, pg/mL	0.547	< .001
eGFR, mL/min/1.73 m^2^	-0.132	0.232
Total Protein, g/dL	-0.117	0.287
Albumin, g/dL	-0.259	0.018
Uric acid, mg/dL	0.242	0.032
LDH, U/L	0.276	0.015
OH, liter	0.405	< .001
OH/ECW	0.435	< .001
ECW, liter	0.266	0.014
ECW/TBW	0.482	< .0001
ICW, liter	-0.153	0.165
TBW, liter	0.083	0.455

### Multiple linear regression analysis

Multiple linear regression analysis was performed for parameters significantly associated with E/e´ ratio except for OH and ECW due to collinearity. Systolic blood pressure, NT-proBNP, and ECW/TBW were independently associated with E/e´ ratio (Table 4).

**Table 4:** Multiple linear regression analysis of variables influencing E/e´ ratio.

	Unstandardized Coefficients		Standardized Coefficients	P-value
	B	Standard Error	Beta	
BPsys, mmHg	0.061	0.028	0.204	0.029
NT-proBNP, pg/mL	0.000	0.000	0.292	0.006
Albumin, g/dL	-0.174	1.026	-0.018	0.866
Uric acid, mg/dL	0.342	0.218	0.146	0.122
OH/ECW	-0.039	0.076	-0.110	0.609
ECW/TBW	49.610	22.118	0.454	0.028

Dependent variable: E/e´ ratio; independent variables: systolic blood pressure, NT-proBNP, albumin, uric acid, OH/ECW, ECW/TBW

### Cut-off values of volume markers

ROC curves were drawn for NT-proBNP, ECW/TBW, OH/ECW, and OH to determine the cut-off values predicting E/e´ ratio greater than 15. The area under curve (AUC) and cut-off values were as follows: 0.73 ± 0.05 (P < 0.001) and 2,797 pg/mL (sensitivity 82.05%, specificity 63.04%) for NT-proBNP; 0.77 ± 0.05 (P < 0.001) and 0.486 (sensitivity 82.05%, specificity 66.96%) for ECW/TBW; 0.73 ± 0.05 (P < 0.001) and 17.28% (sensitivity 71.79%, specificity 68.09%) for OH/ECW; 0.73 ± 0.05 (P < 0.001) and 2.45 liters (sensitivity 74.36%, specificity 63.83%) for OH.

## Discussion

We previously reported that E/e´ ratio predicted mortality and CV events in CKD with the cut-off values of 14.4 and 13.08, respectively [4]. However, the study included a heterogeneous range of CKD patients (CKD stage 3 to 5 and dialysis patients). Although the clinical value of E/e´ ratio in CKD has been emphasized, a more focused approach on a specific subgroup of CKD patients is needed.

Several studies have suggested that uremic cardiomyopathy first begins as diastolic dysfunction, which then develops into progressive fibrosis and ventricular hypertrophy. If this is true, aggressive intervention at early stage could prevent the irreversible cardiac remodeling and poor prognosis associated with it. Therefore, simple clinical assessments to diagnose diastolic dysfunction at an early phase is needed [16, 17]. The patients with advanced CKD have been shown to have progressive increase in LV mass and volume; however, such cardiac remodeling did not affect systolic function whereas diastolic dysfunction was greatly aggravated [18]. Early evaluation of diastolic function is important for CKD patients because diastolic heart failure is associated with higher mortality than systolic heart failure and early diastolic dysfunction is mostly asymptomatic [19, 20]. Since the increased ECW and cardiac remodeling are already evident in early-stage CKD, an early therapeutic approach to correct volume status has been suggested [21].

While NT-proBNP is a clinically valuable biomarker in the diagnosis of heart failure in the general population, it is elevated in CKD patients due to decreased renal excretion [22]. An analysis of patients at high risk of heart failure revealed a significant association between NT-proBNP and LVMI, left atrial size, and E/e´ ratio [23]. However, it is unclear whether NT-proBNP can be used as a marker of cardiac function, specifically diastolic function, in CKD patients. One study has been conducted in a small number of CKD patients, NT-proBNP was not associated with diastolic dysfunction variables; however, the study population was heterogeneous (CKD stage 1 to 5) and the results were not subgrouped according to each CKD stage [24]. Our study found that NT-proBNP was significantly elevated in patients with diastolic dysfunction. Nonetheless, the use of NT-proBNP alone in screening for diastolic dysfunction in CKD patients is limited and no cut-off value for NT-proBNP is provided, suggesting the need for further cardiac imaging [25]. For now, other clinical information must be considered in addition to NT-proBNP in order to reasonably warrant echocardiography in CKD.

E/e´ ratio >15 is generally accepted to reflect abnormal left ventricle relaxation and left ventricular stiffness [26]. Increase in E velocity is mainly related to sodium and water retention, while the decrease in e´ velocity measured by TDI represents increased ventricular stiffness. In patients undergoing hemodialysis, E velocity is elevated before the dialysis session, and then decreases following ultrafiltration due to decreased circulating volume [27]. In contrast, e´ remains similar after ultrafiltration. In other words, e´, which measures the mitral annular diastolic velocity, is relatively unaffected by the patient’s volume status [28]. In our study, LAVI was significantly increased in patients with E/e´ ratio >15. This may be because LAVI and E velocity both reflect volume status. Tsang *et al*. reported that LAVI ≥34 ml/m^2^ distinguished the presence of elevated LV filling pressures (defined by E/e´ ratio >15) with 86% sensitivity and 66% specificity [29]. Our results are in line with that; the cut-off value for LAVI predicting E/e´ ratio >15 was 35.50 ml/m^2^ (AUC 0.78 ± 0.05 [P < 0.001], sensitivity 79.5%, specificity 61.7%). Multiple linear regression analysis investigating on factors correlated with the E/e´ ratio, found that NT-proBNP and ECW/TBW were independently associated. This suggests that NT-proBNP is associated with diastolic dysfunction while also reflecting volume status [8].

Assessment of volume status in CKD patients has become relatively simple and cheap with the development of BIS. However, it is not widely used in clinical practice and echocardiography is most often first performed when clinical symptoms associated with fluid overload (pulmonary edema, cardiomegaly) are already developed. Our results indicate that BIS parameters together with NT-proBNP values could be used to judge whether echocardiographic investigation is warranted (Table 4). Among the parameters of BIS, OH (in liters), OH/ECW (in %), and ECW/TBW can be used as measures of volume status. An OH/ECW of 15% is thought to represent approximately 2.4 liters of overhydration and is used as a cut-off value for overhydration. OH and OH/ECW may also be used to evaluate the risk of diastolic dysfunction, the cut-off value predicting E/e´ ratio >15 was 2.45 liters and 17.28%, respectively. However, in the multiple regression analysis, only ECW/TBW was an independent predictive factor for LV diastolic dysfunction whereas OH/ECW was not. Further investigations including more patients over a longer period of time should focus on developing more accurate methods of diastolic function assessment to confirm these cut-off values.

This study includes several limitations. First, this was a single-center study that included a relatively small number of patients. Also, a full workup on coronary artery disease was not performed, and NT-proBNP values greater than 35,000 pg/mL could not be measured accurately. The duration of diastolic dysfunction could not be analyzed. Despite these limitations, our study excluded patients with LVEF less than 45% and included only patients with stage 5 CKD who had not begun hemodialysis. We also objectively assessed the volume status of the patients at the time of echocardiography with BIS. To our knowledge, this is the first study to have attempted such analysis.

Taken together, asymptomatic diastolic dysfunction is common in patients with stage 5 CKD and our results indicate that BIS parameters and NT-proBNP may be used to select patients needing echocardiography investigation. Thus, these measures may help to diagnose diastolic dysfunction needing intervention, such as pharmacological treatment or correction of volume status, and may result in improved echocardiographic parameters and cardiac prognosis.

## Abbreviations

BPdia: diastolic blood pressure
BPsys: systolic blood pressure
ECW: extracellular water
eGFR: estimated glomerular filtration rate
ICW: intracellular water
LA: left atrium
LAVI: left atrium volume index
LVEDD: left ventricular end-diastolic dimension
LVEDV: left ventricular end-diastolic volume
LVMI: left ventricular mass index
LVEF: left ventricular ejection fraction
OH: overhydration
TBW: total body water
